# Remediation of iron oxide bound Pb and Pb-contaminated soils using a combination of acid washing agents and l-ascorbic acid†

**DOI:** 10.1039/d0ra05327a

**Published:** 2020-10-13

**Authors:** Quan Li, Yilian Li, Zhe Yang, Xiang Li, Zhi Tang, Sen Yang, Yangyang Zhang, Danqing Liu

**Affiliations:** School of Environmental Studies, China University of Geosciences 68 Jincheng Street Wuhan 430074 P. R. China

## Abstract

Soil washing is an efficient, rapid, and cost-effective remediation technique to dissolve target pollutants from contaminated soil. Here we studied the effects of leaching agents: hydrochloric acid (HCl), ethylenediamine tetraacetic acid disodium salt (Na_2_EDTA) and citric acid (CA), and reductants: hydroxylamine hydrochloride (NH_2_OH·HCl) and l-ascorbic acid (VC) on the leaching of Pb from synthetic iron oxide; the changes in mineralogy, morphology, and occurrence of Pb were shown by XRD, SEM, and sequential extraction analyses. Although the washing efficiency of Pb follows the trend HCl (44.24%) > Na_2_EDTA (39.04%) > CA (28.85%), the cooperation of the leaching agent with reductant further improves the efficiency. VC is more suitable as a reductant considering the higher washing efficiency by HCl-VC (98.6%) than HCl–NH_2_OH·HCl (88.8%). Moreover, increasing the temperature can promote the decomposition and dehydrogenation reaction of VC with more H^+^. Among the mixture agents, Na_2_EDTA + VC is the most effective agent to remediate the two kinds of contaminated soils owing to the formation of Fe(ii)–EDTA, a powerful reducing agent so that the efficiencies can reach up to 98.03% and 92.81%, respectively. As a result, these mixture agents have a great prospect to remediate Pb-contaminated soils.

## Abstract 2

In this study, the reductants and time–temperature dependency of dissolved FeO_*x*_–Pb were studied experimentally.

## Graphical abstract

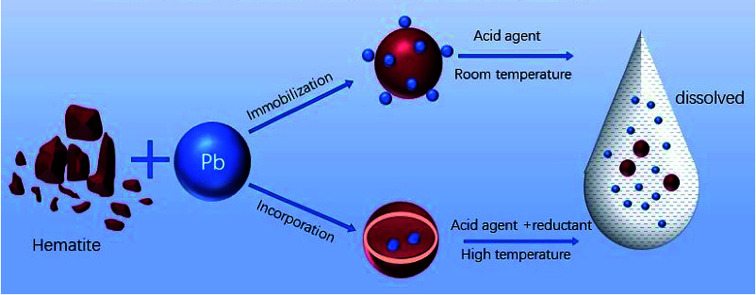

## Introduction

Given the fact about the continuous acceleration of the urbanization process in the 21st century, it is more challenging to solve a series of problems such as environmental pollution, in which the heavy metal contamination is attracting significant attention around the world, including China.^1–3^ Hereinto, lead (Pb) is the 2^nd^ element in the ranking of priority for its long-lasting toxicity.^4^ Pb's high levels can affect the nervous system, kidneys, liver, reproductive system and basic cell processes.^5,6^ Thus, it is urgent to remediate Pb contaminated soils.

Soil washing, a procedure that allows extracting Pb in soils,^7^ is one of the most effective methods because of its facile implementation and short running times.^8^ Numerous adsorption and desorption experiments have shown that using (1) inorganic acids: hydrochloric acid (HCl),^9–11^ nitric acid (HNO_3_)^12^ and sulfuric acid (H_2_SO_4_),^13^ (2) chelating agents: ethylenediamine tetraacetic acid disodium salt (Na_2_EDTA),^9^ ethylenediamine tetraacetic acid (EDTA),^12,14–16^ diethylene triamine pentaacetic acid (DTPA),^17^ ethylenediaminedisuccinic acid (EDDS)^14^ and glutamic diacetic acid (GLDA),^18^ or (3) organic acids: citric acid (CA),^15,16,19^ oxalic acid (OA)^13,15,16^ and maleic acid (MA)^15,20^ is an effective method for the remediation of Pb contamination. In the three types of washing agents, we chose one as the agent to explore the removal of Pb.

Overall, the washing efficiency of Pb depends on agents and contamination characteristics, as well as on the variety of soil physicochemical properties, *e.g.*, soil clay content, carbonate, iron oxide, organic content, *etc.*^21^ Moreover, the bioavailability of heavy metals is very different in relation to their modes of occurrences.^22^ So the occurrence of Pb in contaminated soil is also a critical factor in determining the effectiveness of the remediation. In Hubei province of China, the studies show that Fe–Mn oxide-bound and residual Pb consist of the main fraction of Pb in soil from industrial areas.^23,24^ Moreover, the proportion of heavy metals binding to Fe–Mn oxide is low but high in residue, especially in soil with low pH and high iron oxide content. Considering the fact that, in *x*PbO–*y*Fe_2_O_3_ system, the different combinations of Pb with Fe oxides may lead to the changes in stability,^25–27^ the hydroxylamine hydrochloride, NH_2_OH·HCl, can effectively reduce Mn oxides and the Fe oxides with weak crystalline, whereas just poses a weak influence on the crystalline ferric. Thus, some Pb firmly integrating with Fe oxide is also a kind of “residue”, which can decompose under the reducing environment and then show a higher activity than the Pb remaining in aluminosilicate, resulting in the release of heavy metals.^28,29^ John E. Van Benschoten *et al.* found that adding a reductant such as NH_2_OH·HCl can release only a small amount of the metal even when the soil is high in iron.^30^ The influence of heavy metals in crystalline iron oxide should be attentive when there is high iron oxide in soils. Choosing the proper agent might strengthen the leach of heavy metals binding to iron oxide. On those bases, it is necessary to take into account the dissolution of iron oxide to make Fe–Mn oxide-bound Pb leach effectively. Lu P. *et al.* indicated that desorbed Pb^2+^*versus* dissolved Fe^3+^ data showed a linear relationship for coprecipitation desorption experiments.^31^ Lin Q. *et al.* proposed that low molecular weight organic acids promoted the formation of labile iron on the hematite surface, resulting in the dissolution of hematite.^32^ The mechanisms on hematite dissolution included proton- and ligand (oxalate)-promoted dissolution as well as dark (ascorbic acid) and photochemical (oxalate) reductive dissolution.^33^ A reducing agent is essential for the dissolution of hematite. Ascorbic acid was found to be the most suitable reducing agent, while citric acid–EDTA–ascorbic acid is an effective dissolution medium.^34^ It is necessary and practical to further define the leaching mechanism of iron oxide bound of Pb. However, the previous studies were usually black-box operations, mostly focusing on the washing efficiency of Pb from natural soil or synthetic soil under different treatment methods, without considering the specific performance of pollutants in different phases.

In this paper, the effects of different acids (HCl, Na_2_EDTA and CA) and reductants [NH_2_OH·HCl and l-ascorbic acid (VC)] on Pb removal from the synthesized iron oxide minerals were studied. The objectives of this study were as follows: (1) select an efficient reducing agent for iron oxide mineral dissolution. (2) Obtain the optimal conditions of washing with reducing agent (VC) and washing agents (HCl, Na_2_EDTA, CA). (3) Reveal the removal mechanism of iron oxide bound of Pb by eluents. (4) Assess the pollution level and remediation effect of Pb in contaminated soils.

## Materials and methods

### Samples and reagents

**Fig. 1:**
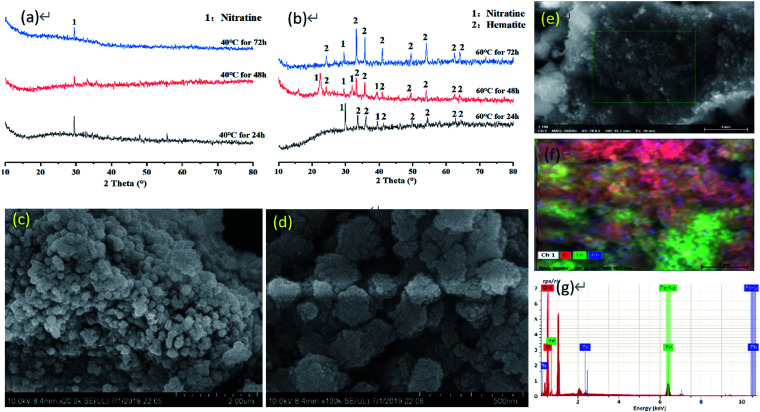
Morphology and chemical composition of the minerals based on XRD, SEM-EDS analysis. (a) 40 °C, (b) 60 °C, (c and d) SEM, (e–g) EDS.

0.05 mol of ferric nitrate, Fe(NO_3_)_3_·9H_2_O, and 0.001 mol of lead nitrate, Pb(NO_3_)_2_, were dissolved in 200 mL ultra-pure water containing 0.152 mol of sodium hydroxide (NaOH) to prepare the iron-oxidation bound Pb and simulate the existence of iron oxide in the soil. To ensure that a large amount of precipitation can be generated as close as possible to the actual soil pH, two samples were adjusted to pH 9 ± 0.05 by NaOH and HNO_3_ then stirred for 24, 48, and 72 h at 40 and 60 °C, respectively, according to the species distribution of Fe^3+^ in Fig. 1s (ESI†). Followed by centrifugation of the suspension at 4000 rpm for 5 min, the samples were washed thrice with ultra-pure water, dried in an oven at 40 °C, pulverized in an agate mortar and sieved by using a #100 mesh (0.149 mm) for subsequent experiments and physiochemical analyses. The total Pb content in samples was measured by inductively coupled plasma mass spectrometry (ICP-MS) after digestion with HF–HNO_3_–HClO_4_.^35^ The two contaminated soils in this study were collected from Wuhan, Hubei Province, China. Roots and rocks were removed and the samples were treated as previously described. Table 1s (ESI†) shows the content of Pb and pH in the soil samples.

## Experiment

The solutions used in this study were: (1) acid washing agents (HCl, Na_2_EDTA, CA) and (2) a mixed solution of acid lotions and reductant (HCl + VC, Na_2_EDTA + VC, CA + VC). To explore the optimum washing conditions, the washing reagents and reductant concentration, liquid–solid ratio and temperature kinetics were investigated using the washing method for repairing iron oxides bound Pb mineral. Mineral (0.100 g) and soils (1.00 g) were mixed with washing solution (20.0 mL), then shaken using a water bath shaker at 220 rpm in 50 mL plastic centrifuge tubes. The suspensions were centrifuged at 4000 rpm for 5 min followed by oscillation and filtration through 0.22 μm membranes. Lastly, the filtrated solution was diluted in 2% nitric acid. The experiments were conducted as follows.

(1) The influences of concentrations of wash agents (HCl and CA: 0.01–0.8 mol L^−1^, Na_2_EDTA: 0.01–0.2 mol L^−1^) on Pb removal were studied over 12 h. (2) The influences of reductants (VC and NH_2_OH·HCl) on Pb removal were studied, depending on the optimum agents (HCl, in step 1). (3) The influences of mixing concentration of acid agents (HCl, CA: 0.01–0.8 mol L^−1^, Na_2_EDTA: 0.01–0.2 mol L^−1^) and reductant (VC: 0.01–0.2 mol L^−1^) on Pb removal were studied, depending on the results in step 2. (4) The influences of other conditions [liquid–solid ratios (10–200) and temperature kinetics (5 min to 12 h) at 25, 40 and 60 °C] on Pb removal were studied, depending on the optimum solution combinations (0.05 M Na_2_EDTA + 0.05 M VC, 0.1 M HCl + 0.1 M VC, 0.1 M CA + 0.08 M VC, in step 3). (5) Investigate the effect on Pb removal from contaminated soils, and compare the changes in the mode of occurrence of Pb before and after washing.

Here, a specific protocol was set out in ESI (Table 2s†). The washing efficiency of the Pb was calculated as follows:1
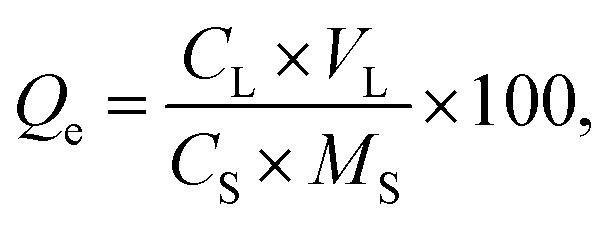
where *Q*_e_ is the Pb washing efficiency, *C*_L_ is the concentration of Pb in the supernatant (μg mL^−1^), *V*_L_ is the volume of washing solution (mL), *C*_S_ is the initial concentration of Pb in the mineral (μg g^−1^) and *M*_S_ is the mass of the sample (g).

### Tessier sequential extraction

The Pb speciation in the minerals before and after washing was performed based on the Tessier sequential extraction procedure.^22^ The Pb was divided into five fractions: exchangeable, carbonate bound, iron and manganese oxides bound, organic matter bound and residual. The extraction procedure was as follows: (i) the exchangeable fraction was extracted with 1 M MgCl_2_ at 25 °C (pH 7.0). (ii) The carbonate bound fraction was extracted with 1 M NaOAc mixed with acetic acid by adjusting the pH to 5.0 at 25 °C. (iii) The iron and manganese oxides bound fractions were extracted with 0.04 M of NH_2_OH·HCl at 25% (v/v) HOAc at 96 ± 3 °C. (iv) The organic matter bound fraction was extracted using 0.02 M of HNO_3_ and 30% H_2_O_2_ by adjusting the pH to 2.0 at 85 ± 3 °C, and then extracted with 3.2 M NH_4_OAc in 20% (v/v) HNO_3_ at 25 °C. (v) The residual fraction was digested with a mixture of HNO_3_–HF–HClO_4_.^35^ After each step, the supernatant was collected and the content of Pb was determined by ICP-MS.

### Morphology and mineralogy of samples

The synthetic mineral samples were identified by X-ray diffraction (XRD) (D8-FOCUS, Bruker Company, Germany) with Cu Kα radiation before and after washing with 0.4 M HCl, 0.05 M Na_2_EDTA and 0.4 M CA for 12 h at a solid/liquid ratio of 1 : 200. The diffractometer was operated at 40 kV and 40 mA with a 2*θ* scan range from 5° to 70° and a step size of 0.02°. The scan speed was of 0.3 s per step. Phase identification was performed with the Eva XRD Pattern Processing software by matching the results with the power diffraction database published by the ICDD.

The morphology, crystallization and coating of the synthetic mineral samples were analyzed by scanning electron microscopy (SEM) (SU8010, Hitachi, Japan) after vacuum gold plating using a 5 kV acceleration voltage. The system was coupled to a backscattered-electron detector for energy-dispersive spectroscopy (EDS).

### Quality assurance

The control treatment involved washing with ultra-pure water. The number of parallel samples accounted for at least 30% of the total sample to ensure the reliability of the data. The quality assurance of the sequential extraction analysis included a blank and specified standardized samples for every 20 normal samples. Additionally, the stability of ICP-MS was tested in accordance with the specifications of internal standards, all of which were met at an acceptable range (80–120%) during operation. All reagents were purchased from Sinopharm Chemical Reagent Co., Ltd. Ultrapure water was obtained from a TKA GenPure system and was used for all experiments and analysis.

## Results and discussion

### Selection of synthetic minerals

The morphology and chemical composition of the minerals as imaged *via* XRD and SEM-EDS is shown in Fig. 1. According to the results of XRD, the main peak of nitratine (at 2*θ* = 29.5°) indicated that there was no crystal of iron oxide under the temperature of 40 °C. However, increasing the temperature to 60 °C promoted the formation of hematite. The longer the reaction time, the better the crystal of hematite. With the time of 72 h at 60 °C, the diffraction peaks of hematite were observable at 2*θ* = 24.2°, 33.2°, 35.8°, 41.0°, 49.7°, 54.2°, 62.5° and 64.0°. But, except for an individual peak at 2*θ* = 29.5°, little nitratine was found. This evidenced that the synthesized minerals presented good crystallinity at 60 °C for 72 h. Hence, we selected the synthetic mineral obtained with these conditions. The results of SEM-EDS on the morphology, crystallization and coating of the mineral produced at 60 °C for 72 h showed that the mineral particles were too small to be observed clearly due to the size of the iron particles. The minerals displayed a nano-spherical structure with significant agglomeration and a uniform distribution of Pb.

The synthetic mineral was used for the sequential extractions of iron exchangeable (S1), carbonates-bound (S2), Fe–Mn oxide-bound (S3), organic matter-bound (S4) and remaining in residue fractions (S5). The Fe–Mn oxide-bound Pb and remaining in residual accounted for 60.5% and 36.4% of the total constitute Pb occurrence in the synthetic mineral, respectively (Table 3s, ESI†). Compared with the content of S3 of Pb in the minerals, the total content of S1, S2 and S4 was 3.15%. The small amount of organic Pb might be due to not washing in the previous step. The quantity of the remaining Pb in the crystalline structure (co-precipitation with crystalline iron minerals) was responsible for the high content of Pb in S5. The Pb in occurrences of S1 and S2 is the most mobile while in S3 and S4 is potentially mobile and in S5 is the most recalcitrant.

### Washing agents and concentrations on Pb washing efficiency

The profile of heavy metals in soil is an important factor in the selection of remediation technologies.^36^ The content of heavy metals in different forms can be varied by adding an appropriate acid or chelating agent to the soil. The purpose of soil washing is to concentrate these metals in the eluate followed by its separation. According to Tessier extraction and analytical results, the proportions of five metal occurrences in the mineral samples were different.

**Fig. 2:**
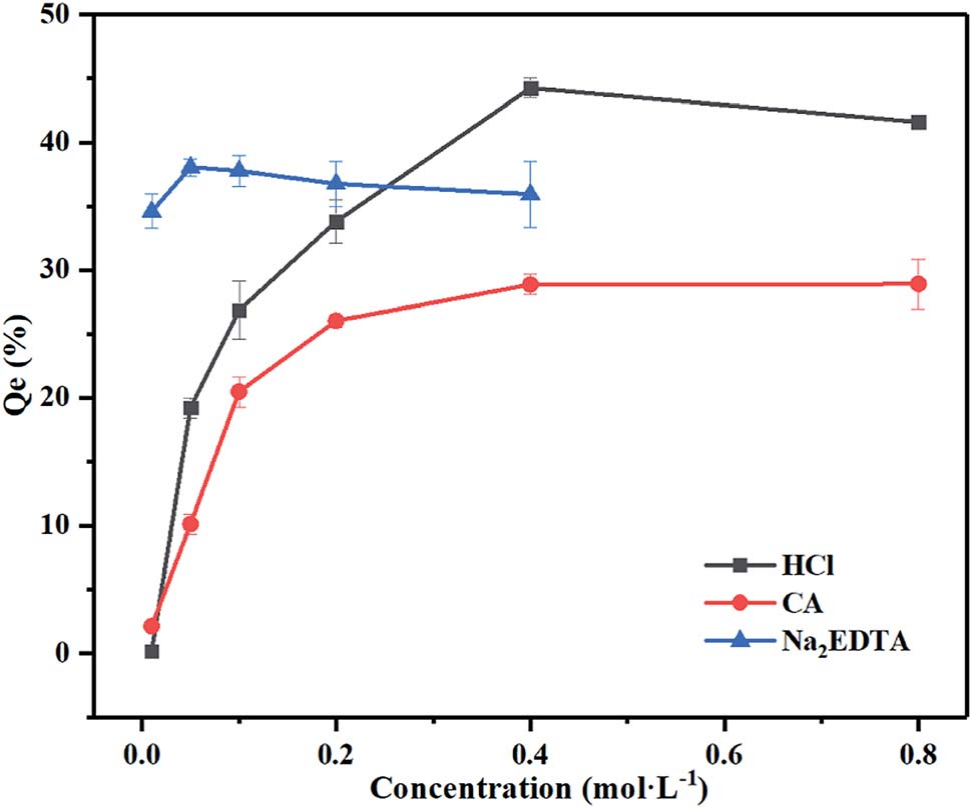
Effects of different washing reagent concentrations on washing efficiency of Pb in iron oxide mineral. Experimental conditions: washing time: 12 h, liquid–solid ratio: 200, temperature: 25 °C.

The results of the washing experiments of iron-oxide-bound Pb mineral are shown in Fig. 2 and are based on three types of single washing agents. Washing with ultrapure water had no effect on Pb removal, indicating that water alone can hardly solubilize Pb from the contaminated mineral.

Regarding the 0.4 M HCl reagent, the maximum desorption rate of Pb from the mineral was 44.2%, with 5.20% and 15.4% increases in the washing efficiency when compared with 0.05 M Na_2_EDTA and 0.4 M CA. The HCl and Na_2_EDTA were more effective than CA in removing Pb. The optimal concentrations of HCl, Na_2_EDTA and CA to remove Pb were found to be 0.4, 0.05 and 0.4 mol L^−1^, respectively. The leaching mechanism of HCl consists on HCl releasing positively charged hydrogen atoms and forming multi-stage protons through H^+^ or the replacement reaction of acid with metals^37^ and dissolved with mineral crystal lattices by the H^+^ absorbed to the surface of minerals.^38,39^ The Na_2_EDTA with Pb can form heavy metal complexes and be adsorbed on the surface of soil particles. The CA can reduce the surface tension between soil particles and heavy metals, therefore rendering heavy metals easier to wash out. Furrer and Stumm (1986) proposed a model of ligand-promoted mineral dissolution that consisted of three steps: (1) organic ligands adsorb on the iron-bearing mineral surface and form complexes with Fe on the surface by ligand exchange (a precursor to dissolution). (2) The complexes detach from the mineral surface. And (3) hydroxylation of Fe on the mineral surface.^40^ The amount of ligand adsorbed, the structure and stability of the complexes are the main factors affecting the process of ligand-promoted mineral dissolution. Hence, the structure of EDTA–Fe(iii) and CA–Fe(iii) complexes affects the mineral dissolution. The CA forms a bidentate surface complex with a stable 5-membered heteromolecular organic ring but with less stability due to the longer carbon chain connecting carboxylic groups.^32^

**Fig. 3:**
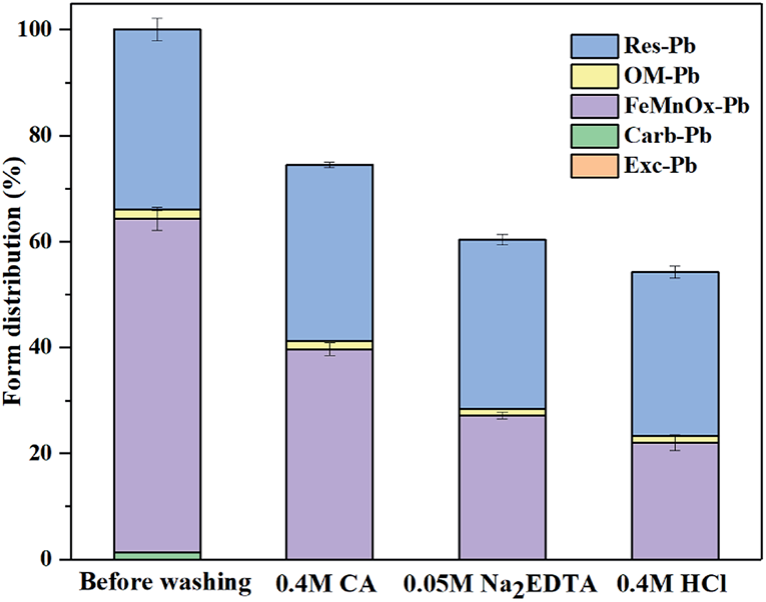
The form of Pb before and after washing with different agents. Experimental conditions: liquid–solid ratio: 200, washing time: 12 h, temperature: 25 °C.

**Fig. 4:**
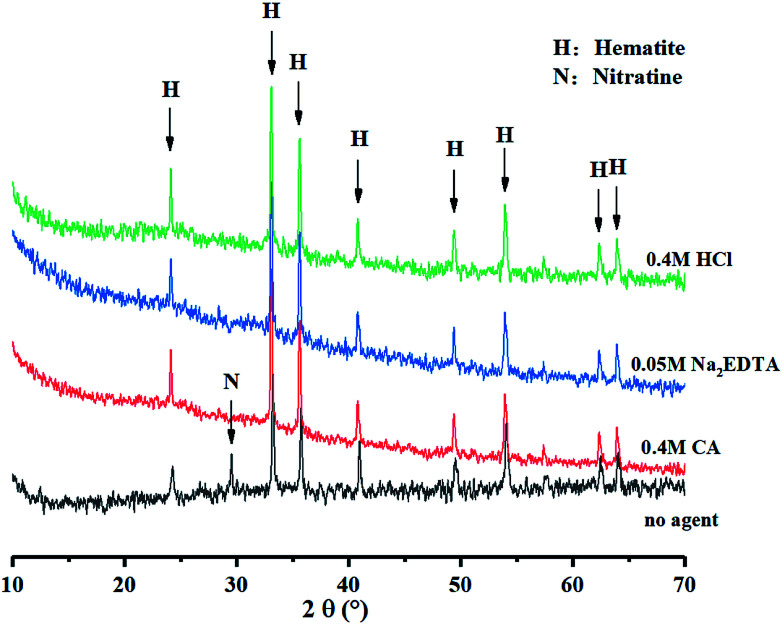
XRD spectra of minerals after washing with different agents.

When remediating Pb-contaminated mineral *via* the washing technique, it is equally important to document the changes in mineral properties. After elution with 0.4 mol L^−1^ HCl, CA and 0.05 mol L^−1^ Na_2_EDTA for 12 h at a liquid–solid ratio of 200 : 1, the minerals were washed with ultrapure water thrice before drying. The samples were subsequently filtered with a #100 mesh for Tessier sequential extraction and XRD analysis. According to Fig. 3, the large proportion of iron oxide-bound Pb was removable, however, there was little material to remove in other occurrences. Fig. 4, shows the XRD pattern, revealing that the peak of 3.02 Å sodium saltpeter at 2*θ* = 29.5° disappeared. This might be due to the dissociated NaNO_3_ seldom precipitating as NaNO_3_ crystals for a high solubility. Moreover, the mineral did not dissolve completely because of the remaining minerals in the residues. Increasing the concentration of agents did not improve the washing efficiency of Pb, indicating that the mineral requires further treatment.

### Comparison of reducing agents with addition of HCl on Pb washing efficiency

The low removal efficiencies of Pb by the three agents were mainly due to the high content of crystalline iron oxides found in synthetic minerals, which is more difficult to decompose than amorphous iron oxide. Therefore, to investigate the leaching effect of iron oxide, an appropriate reducing agent should be mixed with the agents. According to our experimental results, the HCl with the highest washing efficiency was used as the initial agent for subsequent experiments. Two reducing agents, VC and NH_2_OH·HCl, were chosen for the following experiment. The experimental conditions and results are found in ESI (Table 4s†).

The influence of the concentrations of VC, NH_2_OH·HCl and HCl was influential in the desorption behavior of Pb. Adding reducing agent can improve the washing efficiency, with different combinations of reagents delivering distinct effects. The optimal reducing agent was VC, this is likely because the reducibility of VC is stronger than that of NH_2_OH·HCl. When the concentration of VC was 0.05 M and HCl was 1 M, or when VC was 0.1 M and HCl was 0.4 M, the Pb was completely removable owing to the considerable dissolution of defects and edges in minerals.^41^ Adding VC to HCl can increase the Pb washing efficiency because the lattice Fe(iii) ions are reduced in the presence of a reducing agent and the formed Fe(ii) ions can no longer be at the original Fe(iii) sites because of their larger sizes.^34^ The H^+^ can then be adsorbed in the bonding sites of the iron oxide surface, contributing to the dissolution of iron oxide as well as the removal of Pb.^42^

### Optimal mixed agents and concentrations on Pb washing efficiency

**Fig. 5:**
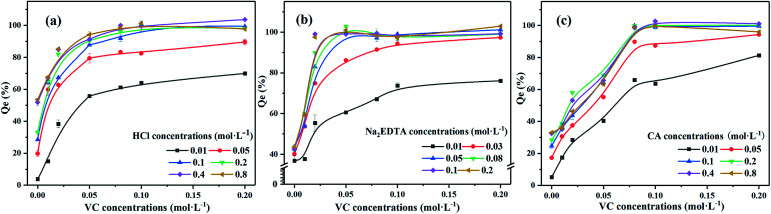
Effects of mixture agent concentrations on Pb washing efficiency. Experimental conditions: liquid–solid ratio: 200, washing time: 12 h, temperature: 60 °C. (a) HCl + VC, (b) Na_2_EDTA + VC, (c) CA + VC.

We found that VC was the optimal reductant. Next, we investigated the influence of eluting agents on the Pb washing efficiency with the existence of VC, the results of which are displayed in Fig. 5. Removal efficiencies of Pb were nearly 100% with 0.1 M HCl + 0.1 M VC, 0.05Na_2_-EDTA + 0.05 VC and 0.1 M CA + 0.08 M VC, which indicates that the concentration of agents were appropriate due to the incomplete removal of contamination with low concentration or the surplus of agents with high concentration. In addition, compared with washing agents without reductant, the iron oxide mineral was completely dissolved and the lead was thoroughly removed upon adding a reductant. Although VC is a powerful reducing agent, it is not a good complexing agent. Na_2_EDTA proved to be a much better complexing agent than CA or VC. The reaction between the reducing agent and the crystalline iron oxide could promote the transformation of Fe^3+^ into Fe^2+^. Moreover, the Fe(ii)–EDTA formed in the solution is a powerful reducing agent and the dissolution rates increased faster with increasing Na_2_EDTA concentrations than in HCl and CA.^34^ This can help to accelerate the dissolution of the mineral and promote the release of Pb^2+^. Meanwhile, adding reductant can also reduce the amount of acid and greatly improve the washing efficiency of Pb. For the polluted soil with high content of iron and manganese oxidation occurrence of Pb, a proper amount of reducing agent can thus improve the effectiveness of Pb-contaminated soil remediation.

### Effect of liquid–solid ratio on Pb washing efficiency

**Fig. 6:**
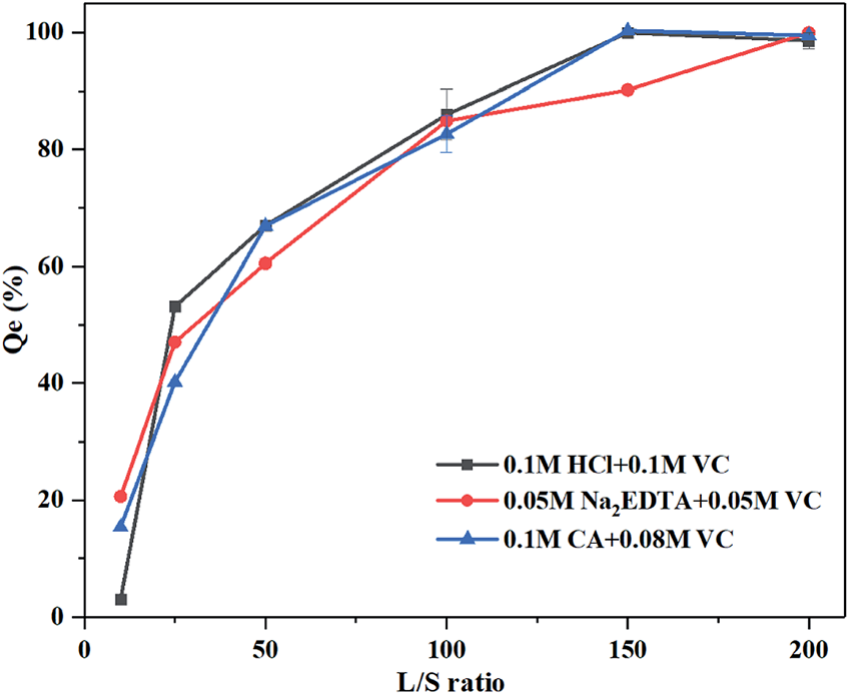
Effect of liquid–solid ratio on Pb washing efficiency. Experimental conditions: washing time: 12 h, temperature: 60 °C. Concentrations of mixed washing agents: (1) 0.1 mol L^−1^ HCl + 0.1 mol L^−1^ VC, (2) 0.05 mol L^−1^ Na_2_EDTA + 0.05 mol L^−1^ VC, (3) 0.08 mol L^−1^ CA + 0.1 mol L^−1^ VC.

The three mixed agents showed similar trends in the removal of Pb, with a rapid increase followed by stabilization (Fig. 6). The washing efficiency was 100% for 0.05 M Na_2_EDTA + 0.05 M VC at a liquid–solid ratio of 200, and for 0.1 M HCl + 0.1 M VC and 0.1 M CA + 0.08 M VC at a liquid–solid ratio of 150; the mineral was dissolved completely. Thus, it is possible to remediate iron oxide-bound Pb contaminated soil by employing this agent mixture with an acid and reductant.

### Temperature kinetics of Pb extraction by washing agents with addition of reductant agents

**Fig. 7:**
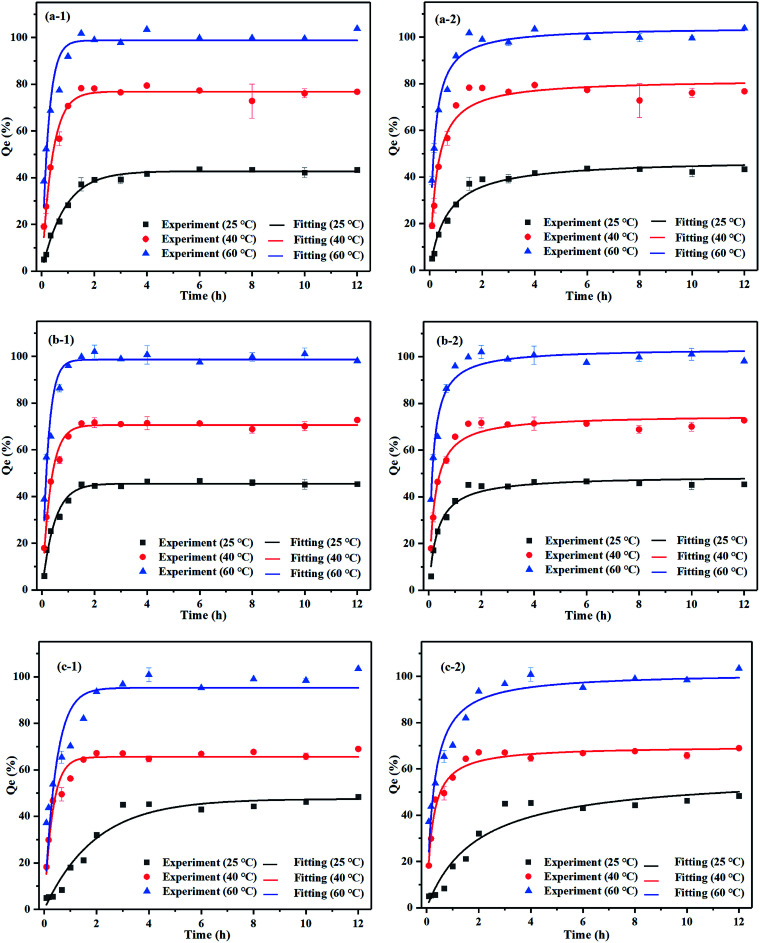
Kinetics of Pb. Experimental conditions: mixture agent concentration and liquid–solid ratio: (a) 0.1 M HCl + 0.1 M VC, 150, (b) 0.05 M Na_2_EDTA + 0.05 M VC, 200, (c) 0.1 M CA + 0.08 M VC, 150. (x-1) Pseudo-first-order equation. (x-2) Pseudo-second-order equation.

In terms of the effect of reducing agents on removal efficiencies, we found the best combinations to be mixtures of agents with 0.1 M HCl + 0.1 M VC, 0.05 M Na_2_EDTA + 0.05 M VC and 0.1 M CA + 0.08 M VC. Regarding the Pb washing efficiency, the temperature kinetics from 25 °C to 60 °C were studied under the experimental conditions of the mixture agents at optimum liquid–solid ratios. We observed that upon raising the temperature, the kinetics were excellent in terms of the temperature dependence. Both temperature and time play an important role in the washing efficiency of iron oxide bound of Pb.^43^Fig. 7 shows that the Pb leaching efficiencies increased notoriously with raising the temperature to 60 °C. Also, the mineral was dissolved completely in contrast with the temperatures of 25 °C and 40 °C. The temperature was thus observed to be an important trigger for the reactions including the desorption of Pb^2+^ from the mineral particles and some chemical reactions between the leaching agent and Pb-bearing minerals. For three mixture agents, the mineral washing process was divided into three time periods in which the washing efficiency of Pb was increased rapidly, grew steadily, and finally stabilized as the time passed. The higher the temperature the faster the reaction efficiency and the higher the washing efficiency of Pb. The maximum desorption rate of Pb was almost 100% at 60 °C and the mineral was dissolved completely by those mixture agents. Because VC contains an unstable ene glycol structure, oxygen and VC molecules tend to be more active upon increasing the temperature, which promotes the decomposition of VC with more H^+^ and accelerates the dehydrogenation reaction speed of VC. Meanwhile, with the increase in temperature, the washing efficiency of VC to superoxide anion radical decreases, accelerating its decomposition. The process in which VC breaks down to produce colored substances turning the supernatant dark brown is known as nonenzymatic browning reaction. For HCl + VC and Na_2_EDTA + VC the reaction reaches equilibrium after 2 h. On the other hand, the CA + VC reaches equilibrium at 60 °C for 3 h.

Kinetic data were fitted into pseudo-first-order and pseudo-second-order models to describe the process of Pb washing efficiency from iron oxide mineral to mixture washing agents.

The pseudo-first-order equation being

2 *Q*_*t*_ = *Q*_1_(1 − e^−*k*_1_*t*^)

and the pseudo-second-order equation being3
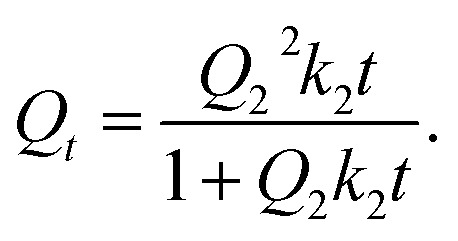
where *Q*_1_ and *Q*_2_ are the removal efficiencies of Pb (%) at equilibrium, *Q*_*t*_ is the washing efficiency of Pb at time *t* (%) and *k*_1_ and *k*_2_ are the equilibrium rate constants.

The pseudo-first-order model applies to the initial kinetic process while pseudo-second-order describes the whole process of desorption. The *R*^2^ values indicated that Pb washing efficiency to mixture agents was better described by the pseudo-second-order model, which is indicative of the chemical desorption process. The parameters of kinetics can be found in ESI (Table 5s†).

### Exploration of practical use

Regarding the three mixture agents, although HCl was detected on soil properties and bioavailability during the washing processes, this is often used for chemical leaching at large scale. Because HCl is one of the least hazardous strong acids commonly used, despite its acidity, it consists of the non-reactive and non-toxic chloride ion.^44^ The negative effects of Na_2_EDTA on biodegradability have also been reported, although it possess the ability to form stable metal complexes^45^ while also offering the possibility to be recuperated and recycled.^46^ Citric acid is a small, easily biodegradable organic acid, therefore, it is widely used as an environmentally friendly agent to remediate contaminated soil.^47^

**Fig. 8:**
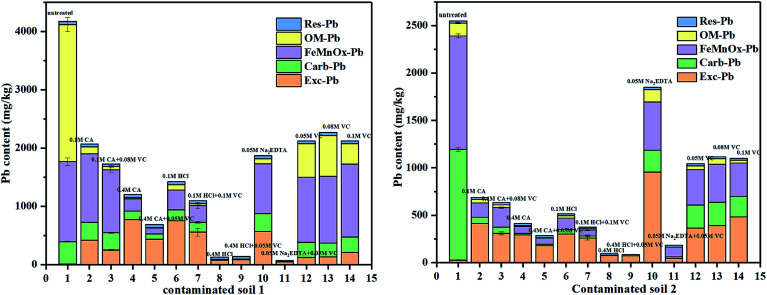
Influence on the Pb content in contaminated soils after different treatments. Experimental conditions: washing time: 12 h, temperature: 60 °C, washing agents concentrations and liquid–solid ratio: ((1) untreated soil; (2) 0.1 mol L^−1^ CA; (3) 0.1 mol L^−1^ CA + 0.08 mol L^−1^ VC; (4) 0.4 mol L^−1^ CA; (5) 0.4 mol L^−1^ CA + 0.05 mol L^−1^ VC; (6) 0.1 mol L^−1^ HCl; (7) 0.1 mol L^−1^ HCl + 0.1 mol L^−1^ VC; (8) 0.4 mol L^−1^ HCl; (9): 0.4 mol L^−1^ HCl + 0.05 mol L^−1^ VC; (10) 0.05 mol L^−1^ Na_2_EDTA; (11) 0.05 mol L^−1^ Na_2_EDTA + 0.05 mol L^−1^ VC; (12) 0.05 mol L^−1^ VC; (13) 0.08 mol L^−1^ VC; (14) 0.1 mol L^−1^ VC). The experimental conditions for sample 1, and 2 were listed in the ESI.†

The experimental conditions of the practical soil washing are listed in ESI (Table 6s†). The Pb washing efficiency can be found in Table 1. Modes of occurrence of Pb in soils are shown in Fig. 8. The amount of desorbed Pb varied with the different washing agents and increased with increasing the concentration of washing agents. Addition of CA and VC firstly resulted in a decreased concentration of organic matter bound of Pb but a considerable increase in the iron exchangeable Pb, which is the most available portion in soil for plant nutrient uptake and can exert harmful effects in living organisms. Furthermore, the addition of VC had a positive effect on decreasing the concentration of Pb with CA, HCl and Na_2_EDTA. Meanwhile, the HCl had a positive effect on the extraction of all occurrences of Pb in contaminated soils. The noticeable positive correlation between the exchangeable Pb and acid washing agents was possibly because Pb can form strong bonds with these agents. On the contrary, the addition of VC lead to a significant decrease in the concentration of Pb with Na_2_EDTA, this is because Na_2_EDTA is alkaline, which is not favorable to the desorption of Pb in the soil. After the addition of VC, the acidified Na_2_EDTA can effectively remove Pb from the soil, unlike the desorption of Pb in iron oxide mineral, this may be related either with the degree of crystallization of the iron oxide in the soil or with the influence of organic and other compounds in soil on the desorption of lead. It is well known that NH_2_OH·HCl has an obligate solution-extraction effect on heavy metals in Fe–Mn oxidation occurrence,^48^ whereas VC has a low effect of obligatory solute. As shown in Fig. 8, the addition of VC had a great impact on the organic occurrence of Pb, this is because VC is not only a reducing agent but also an organic acid which can react with the organic compounds in the soil, leading to the removal of organic occurrence of Pb in the contaminated soils.

**Table 1:** Pb washing efficiency and the supernatant pH after washing

No.	Agent concentration	Contaminated soil 1			Contaminated soil 2		
		Pb content after washing (mg kg^−1^)	Pb washing efficiency (%)	Supernatant pH	Pb content after washing (mg kg^−1^)	Pb washing efficiency (%)	Supernatant pH
2	0.1 M CA	2069	50.4	3.34	685	73.1	3.83
3	0.1 M CA + 0.08 M VC	1727	58.6	3.52	634	75.1	4.11
4	0.4 M CA	1204	71.1	2.30	413	83.8	2.26
5	0.4 M CA + 0.05 M VC	687	83.5	2.48	284	88.9	2.10
6	0.1 M HCl	1421	65.9	2.25	515	79.8	1.41
7	0.1 M HCl + 0.1 M VC	1093	73.8	2.48	375	85.3	2.48
8	0.4 M HCl	128	96.9	0.92	94.0	96.3	0.92
9	0.4 M HCl + 0.05 M VC	138	96.7	0.97	85.0	96.7	0.97
10	0.05 M Na_2_EDTA	1866	55.3	2.89	1847	27.6	3.24
11	0.05 M Na_2_EDTA + 0.05 M VC	71.0	98.3	4.46	183	92.8	4.25
12	0.05 M VC	2123	49.1	4.44	1043	59.1	4.68
13	0.08 M VC	2266	45.7	4.22	1118	56.2	4.39
14	0.1 M VC	2124	49.1	4.05	1101	56.8	4.16

Compared with other solutions, 0.4 mol L^−1^ CA + 0.05 mol L^−1^ VC, 0.4 mol L^−1^ HCl and 0.05 mol L^−1^ Na_2_EDTA + 0.05 mol L^−1^ VC were appropriate to remove the Pb in soil 1 while 0.1 mol L^−1^ CA, 0.1 mol L^−1^ HCl and 0.05 mol L^−1^ Na_2_EDTA + 0.05 mol L^−1^ VC were appropriate to treat soil 2. Following treatment, the Pb content was acceptable according to the Chinese Soil Environmental Quality for Construction Land Standard Two (800 mg kg^−1^).

The pH of the soil before and after washing is shown in Table 1. We observe that, although HCl has a good washing efficiency of soil lead, it had a significant influence on soil pH. On the contrary, the Na_2_EDTA + VC mixture had a high washing efficiency of soil lead and had little influence on the soil pH. In addition to HCl, which is the standard agent for soil re-use after washing, CA and VC can also achieve the washing efficiency of Pb. However, CA and Na_2_EDTA mixed with VC were considered to exert the least acidification and therefore were found to be the most environmentally friendly alternative.

## Conclusion

The leaching behaviors of Fe oxide-bound Pb from synthetic iron oxide minerals and soils were analyzed through washing and sequential extraction methods. We conclude that:

(1) With the initial pH of 9, the optimum condition for synthesis Fe oxide-bound Pb was at 60 °C for 72 h.

(2) There was a significant synergistic effect between washing agents and VC, in which the optimum condition for Pb leaching from synthetic mineral was the mixture of 0.1 M HCl + 0.1 M VC for 2 h, 0.05 M Na_2_EDTA + 0.05 M VC for 2 h and 0.1 M CA + 0.08 M VC for 3 h at 60 °C, respectively. Furthermore, higher temperatures were demonstrated to have a positive effect on Pb washing.

(3) The addition of VC had the synergistic effect on decreasing Pb concentration with washing agents, especially on Na_2_EDTA. The 0.4 mol L^−1^ CA + 0.05 mol L^−1^ VC, 0.4 mol L^−1^ HCl and 0.05 mol L^−1^ Na_2_EDTA + 0.05 mol L^−1^ VC were the best washing agents for contaminated soil 1 and 0.1 mol L^−1^ CA, 0.1 mol L^−1^ HCl and 0.05 mol L^−1^ Na_2_EDTA + 0.05 mol L^−1^ VC were the best washing agents for contaminated soil 2. The Pb content was acceptable according to the Chinese Soil Environmental Quality for Construction Land Standard Two.

(4) Although HCl had a negative effect on soil pH, it also showed the best effect on Pb removal. Meanwhile, Na_2_EDTA and CA mixed with VC was also promising for the remediation of Pb-contaminated soils, especially with high content of iron and manganese oxidation occurrence of Pb.

(5) Although the washing agents mixed with VC had an active effect on the remediation the contaminated soil, the effect of VC in Fe–Mn oxidation occurrence and organic occurrence bound Pb should be further investigated.

## Conflicts of interest

There are no conflicts to declare.

## Supplementary Material

RA-010-D0RA05327A-s001
