# Supplementary material for: Remediation of iron oxide bound Pb and Pb-contaminated soils using a combination of acid washing agents and l-ascorbic acid
Source: RSC Adv. 2020 Oct 13;10(62):37808–17. doi: 10.1039/d0ra05327a (PMC9057221; doi:10.1039/d0ra05327a)
Supplement: RA-010-D0RA05327A-s001 [file RA-010-D0RA05327A-s001.pdf]

Electronic Supplementary Material (ESI) for RSC Advances.
This journal is © The Royal Society of Chemistry 2020

Supplementary Materials for

Remediation of Iron Oxide Bound of Pb and Pb-Contaminated Soils Using a Combination of Acid Washing Agents and L-ascorbic Acid

Quan Li,[a] Yilian Li,*[a] Zhe Yang,[a] Xiang Li,[a] Zhi Tang,[a] Sen Yang,[a] Yangyang Zhang,[a] Danqing Liu[a]

a. School of Environmental Studies, China University of Geosciences, 68 Jincheng Street, Wuhan 430074, P. R. China

*Corresponding author: yl.li@cug.edu.cn

4 Pages, 6 Tables, 1 Figures

The form of Fe in solution and mineral

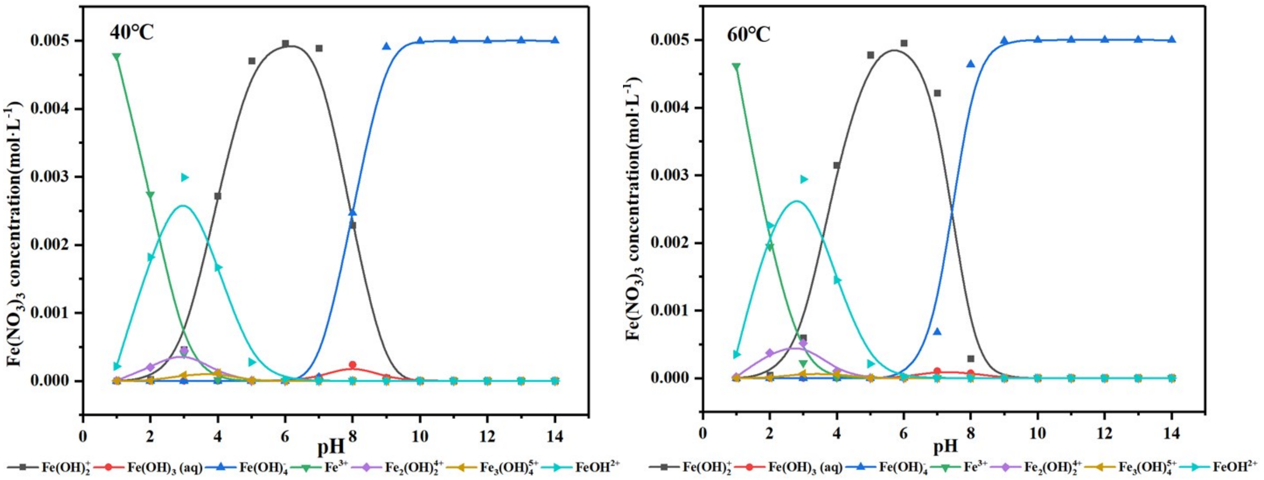

Fig. 1s Calculation results of the Fe(III) species distribution under variable pH conditions. Experiment conditions: concentration: 0.005mol·L^{-1} $Fe(NO_3)_3$, temperature: 40°C on the left, 60°C on the right, atmospheric pressure: 101.325KPa.

Basic property of Pb-contaminated soils

Table 1s The Pb content and pH of the contaminated soils

Contaminated soil no.	Total Pb (mg/kg)	pH
1	4319.3	8.5
2	2544.6	7.13

Experimental conditions

Table 2s The detailed protocol for Pb washing experiments in mineral and soils

Group	Factor					
	Additive variety		Concentration /mol	Liquid-solid ratio	Temperature /℃	Time /h
1	Washing agents	Inorganic acid agent (HCl)	0.01, 0.05, 0.1, 0.2, 0.4, 0.8	20	25	12
		chelating agent (Na_2EDTA)	0.01, 0.05, 0.1, 0.2, 0.4	20	25	12
		organic acid agent (citric acid)	0.01, 0.05, 0.1, 0.2, 0.4, 0.8	20	25	12
2	Reductive agent selection	NH_2OH • HCl + HCl	NH_2OH • HCl: 0, 0.04, 0.1	20	60	12
			HCl: 0, 0.4, 1	20	60	12
		VC + HCl	VC: 0, 0.05, 0.1	20	60	12
			HCl: 0, 0.4, 1	20	60	12

3	Mixed solution	VC + HCl	VC: 0, 0.01, 0.02, 0.05, 0.08, 0.1, 0.2<br>HCl: 0.01, 0.05, 0.1, 0.2, 0.4, 0.8	200	60	12
		VC + Na_2EDTA	VC: 0, 0.01, 0.02, 0.05, 0.08, 0.1, 0.2<br>Na_2EDTA: 0.01, 0.03, 0.05, 0.08, 0.1, 0.2	200	60	12
		VC + CA	VC: 0, 0.01, 0.02, 0.05, 0.08, 0.1, 0.2<br>CA: 0.01, 0.05, 0.1, 0.2, 0.4, 0.8	200	60	12
4	Liquid-solid ratio selection	VC + HCl	VC: 0.1<br>HCl: 0.1	10, 25, 50, 100, 150, 200	60	12
		VC + Na_2EDTA	VC: 0.05<br>Na_2EDTA: 0.05	10, 25, 50, 100, 150, 200	60	12
		VC + CA	VC: 0.08<br>CA: 0.1	10, 25, 50, 100, 150, 200	60	12
5	Temperature kinetics	VC + HCl	VC: 0.1<br>HCl: 0.1	150	25, 40, 60	5 min, 10 min, 20 min, 40 min, 1h, 1.5h, 2 h, 4 h, 6 h, 8 h and 12 h
		VC + Na_2EDTA	VC: 0.05<br>Na_2EDTA: 0.05	200	25, 40, 60	
		VC + CA	VC: 0.08<br>CA: 0.1	150	25, 40, 60	
6	Contaminated soils	VC + HCl	0.1 HCl, 0.1 HCl + 0.1VC, 0.4 HCl, 0.4 HCl + 0.05VC	150	60	12
		VC + Na_2EDTA	0.05 Na_2EDTA, 0.05 VC + 0.05Na_2EDTA	200	60	12
		VC + CA	0.1, 0.4	150	60	12
		VC	0.05, 0.08, 0.1	200	60	12

Table 3s The occurrence of Pb in the minerals (ug/g)

Pb in the Mineral	Exchangeable	Carbonate-bound	Fe-Mn oxides-bound	Organic-bound	Residual	Total content
Pb	0.8	463.09	20052.03	581.79	12046.33	33144.11

Washing experiment conditions and simulated results

Table 4s The two reducing agents and concentrations on Pb removal efficiency

Test NO.	Mineral mass (g)	The volume of the mixed solution (ml)	HCl concentration	Reductant concentration	Temperature (°C)	Removal efficiency (%)	pH of the supernatant after washing
EX-1	0.1	20	0.4 mol·L^{-1} HCl	-	60	53.7	0.56
EX-2	0.1	20	-	0.04 mol·L^{-1} $NH_2OH·HCl$	60	23.6	3.6
EX-3	0.1	20	0.4 mol·L^{-1} HCl	0.04 mol·L^{-1} $NH_2OH·HCl$	60	61.2	0.56
EX-4	0.1	20	1 mol·L^{-1} HCl	0.04 mol·L^{-1} $NH_2OH·HCl$	60	88.8	0.18
EX-5	0.1	20	0.4 mol·L^{-1} HCl	0.1 mol·L^{-1} $NH_2OH·HCl$	60	61.4	0.55
EX-6	0.1	20		0.05 mol·L^{-1} VC	60	40.5	3.77
EX-7	0.1	20	0.4 mol·L^{-1} HCl	0.05 mol·L^{-1} VC	60	90.3	0.62
EX-8	0.1	20	1 mol·L^{-1} HCl	0.05 mol·L^{-1} VC	60	98.6	0.25
EX-9	0.1	20	0.4 mol·L^{-1} HCl	0.1 mol·L^{-1} VC	60	95.7	0.62

Table 5s Parameters of kinetics of Pb removal efficiency from iron oxide mineral to washing agents.

Washing agent	Temperature (°C)	Pseudo-first-order(x-1)			Pseudo-second-order(x-2)		
		Q_1 (%)	k_1($\%^{-1}h^{-1}$)	R_1^2	Q_2 (%)	k_2($\%^{-1}h^{-1}$)	R_2^2
a (0.1M HCl+0.1M VC)	25	42.78	1.1521	0.991*	47.57	0.0321	0.977*
	40	76.85	2.5112	0.980*	81.92	0.0477	0.949*
	60	98.76	3.9374	0.912*	104.34	0.0598	0.969*
b (0.05M Na_2EDTA+0.05M VC)	25	45.52	2.1323	0.980*	49.01	0.0646	0.967*
	40	70.61	3.0720	0.982*	75.07	0.0630	0.969*
	60	98.59	4.3372	0.939*	103.55	0.0687	0.964*
c (0.08M CA+0.1M VC)	25	47.49	0.5176	0.958*	58.05	0.0090	0.935*
	40	65.51	3.1804	0.942*	69.88	0.0683	0.975*
	60	95.21	2.3004	0.805*	101.62	0.0371	0.925*

*Significant at P=0.01 level

Table 6s Experimental conditions of contaminated soils

Group no.	Acid washing agents ($mol·L^{-1}$)	VC ($mol·L^{-1}$)	Time (h)
1-untreated	-	-	-
2	0.1mol·L^{-1} CA	-	12
3	0.1mol·L^{-1} CA	0.08	12
4	0.4mol·L^{-1} CA	-	12
5	0.4mol·L^{-1} CA	0.05	12
6	0.1mol·L^{-1} HCl	-	12
7	0.1mol·L^{-1} HCl	0.1	12
8	0.4mol·L^{-1} HCl	-	12
9	0.4mol·L^{-1} HCl	0.05	12
10	0.05mol·L^{-1} Na_2EDTA	-	12
11	0.05mol·L^{-1} Na_2EDTA	0.05	12
12	-	0.05	12
13	-	0.08	12
14	-	0.1	12
